# Let the peptides shine: SOX (Sulfonamido-OXine)-labelled peptides for direct kinase and phosphatase monitoring

**DOI:** 10.1039/d6cb00048g

**Published:** 2026-05-19

**Authors:** Lydia E. Papagora, Stephen A. Cochrane

**Affiliations:** a School of Chemistry and Chemical Engineering David Keir Building, Stranmillis Road, Queen's University Belfast Belfast BT9 5AG UK s.cochrane@qub.ac.uk

## Abstract

Protein kinases and their cognate phosphatases are central regulators of cellular signalling, acting through the opposing processes of phosphorylation and dephosphorylation. Defects in kinase/phosphatase signalling are implicated in many diseases, including cancer, neurodegenerative conditions and metabolic disorders. Rapid and economical methods for measuring kinase activities are critically needed as diagnostic tools and for the evaluation of kinase inhibitors, including approaches that allow direct and quantitative measurement of kinase activities in complex media like cell lysates. SOX (sulfonamido-oxine)-labelled peptides, that upon phosphorylation chelate Mg^2+^ and undergo Chelation-Enhanced Fluorescence (CHEF), have emerged as a convenient class of modular kinase sensors. The development of an array of SOX-peptides has allowed researchers to gain insights into biological processes including binding events, conformational constraints, enzyme activities, protein trafficking and localization. This focused review provides an overview of the use of SOX-labelled peptides for probing kinase and phosphatase activity in real-time assays, including their development, synthesis, applications and strengths and weaknesses compared to other kinase assays.

## Abstract 2

SOX-labelled peptides enable direct, continuous fluorescence readouts of kinase and phosphatase activity. These CHEF-based probes provide real-time enzymatic assays in purified systems and cell lysates.

## Graphical abstract

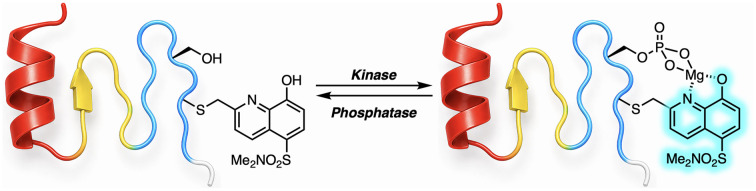

## Introduction

### Common kinase assays and limitations

**Fig. 1:**
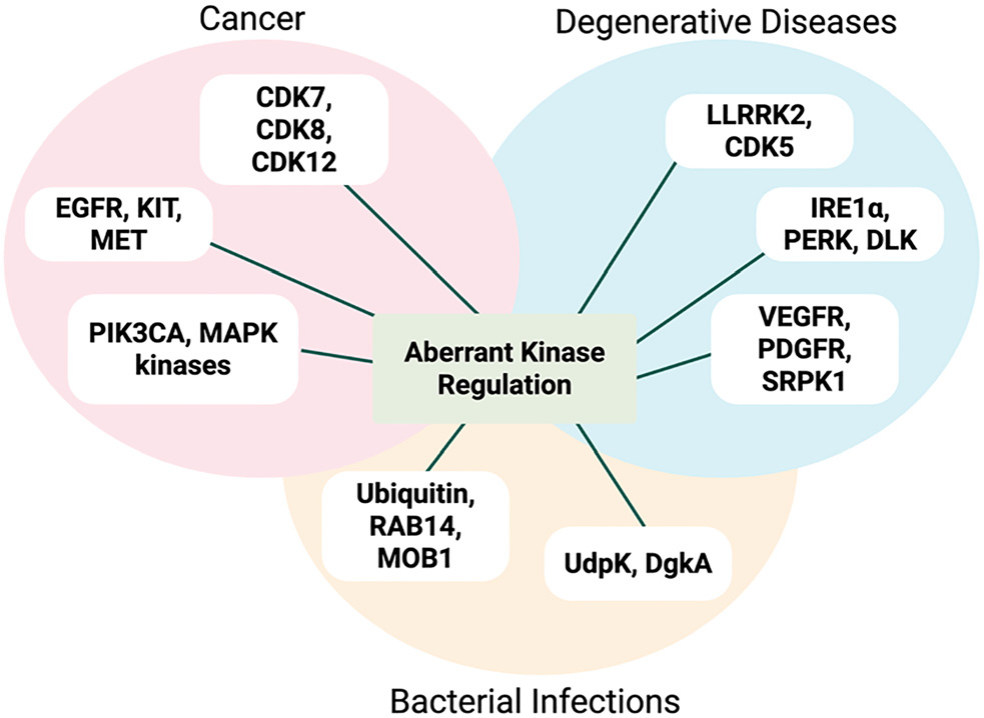
Representative examples of different classes of kinase aberrant activity in many diseases, including cancer, neurodegenerative disorders and bacterial infections.

Protein phosphorylation on Ser, Thr and Tyr is a common post-translational modification (PTM).^1,2^ This PTM is controlled by the opposing actions of kinases and phosphatases, ubiquitous and highly conserved elements of signalling pathways.^2–6^ Although different protein kinases process different substrates, they share broadly conserved structural motifs, including ATP-binding domains and catalytic site residues.^7–9^ Aberrant levels of kinase activity are associated with cancer, infectious diseases, neurodegenerative conditions and metabolic disorders (Fig. 1).^2,8,10,11^ Therefore, detection of kinase activity is crucial for screening novel kinase inhibitors and identification of kinase substrates.^10^ Sensitive, selective and high-throughput chemosensors are invaluable in academic and pharmaceutical settings for unravelling these diverse signalling cascades.^12,13^

The crucial roles of kinases and phosphatases in normal cellular communication and physiology underscore the unmet need for robust analytical tools.^2,14,15^ Functional *in vitro* assays facilitate kinase profiling, evaluating drug efficacy prior to *in vivo* analyses. Typically, phosphorylated substrates can be quantified indirectly or directly using endpoint (discontinuous) or continuous (real-time) assays, respectively.^16,17^ In endpoint assays, an aliquot of the reaction mixture is removed prior to analysis of the reaction product (ADP or phosphorylated substrate). For these assays to be valid, the chosen timepoint must fall in the linear portion of the progress curve so that the endpoint signal reflects the initial reaction rate.^18,19^ While endpoint assays are valuable for early profiling and selectivity screening, they provide limited information on enzyme kinetics and inhibitor mechanism.^12^ Thus, time-dependent inhibition (TDI) can be missed or mischaracterised.^20^ Unlike endpoint methods, real-time assays track reactions over the entire course of an experiment. By capturing full progress curves, continuous assays detect TDI accurately, facilitating the screening of potent inhibitors.^16,17,21^ Moreover, they differentiate between reversible and irreversible inhibition mechanisms in complex biological samples.^22,23^ This is particularly effective for quantifying kinetic parameters and providing PK/PD strategies. Commonly employed approaches for monitoring kinase activity include mass spectrometry-based, immunoassays, and radioactivity-based approaches.^2,24^ While these techniques have proven effective, they also have several limitations.^2,22,25,26^ Quantitative mass spectrometry platforms require sample enrichment to detect low-abundance analytes.^27^ Immunoassays rely on validation of the antibodies from batch to batch to ensure their specificity.^28^^32^P-based assays are inherently discontinuous and hence not practical for high-throughput screening applications.^2,29,30^ Fluorescence sensing is a compelling alternative in biomedical research and clinical applications, offering operational simplicity, rapid response kinetics and suitability for bioimaging with spatiotemporal resolution.^24,31,32^ Various kinds of kinase sensing systems including fluorophore dyes, nanomaterials and lanthanides are used to interrogate signalling processes at the molecular level. Despite these contributions, several technical, biological and material-related concerns remain. Fluorophore dyes (for example Alexa Fluor 647, cyanine dyes and rhodamine derivatives) yield signals that are strongly influenced by photostability, duty cycle, sensitivity to buffer conditions and activation rates by light sources.^33–35^ Additionally, they can introduce cellular perturbations which complicate their application in kinase assays.^36–38^ Fluorescent nanomaterials (FNMs) include gold nanoclusters (AuNCs), carbon nanodots (CDs) and quantum dots (QDs). However, most nanomaterial-based kinase assays are endpoint.^39–42^ In the synthesis process, the surface of FNMs can be hard to distribute uniformly due to agglomeration of small particles. FNMs also suffer from variable quantum yields that compromise quantitative accuracy of assays.^43–47^ Lanthanide-based compounds are being actively developed in biomedical imaging, yet limited progress in kinase sensing has been achieved so far. Surface quenching owing to surface defects, adsorbed water, or hydroxyl groups encourages non-radiative relaxation.^48,49^ Rare-earth elements are associated with high extraction costs and environmentally damaging mining practices. Moreover, most of the advanced synthesis methods are not easily translatable to industrial-scale production.^50,51^ Until now, only a few simple and rapid single step continuous assays have been developed. Förster Resonance Energy Transfer (FRET) assays offer a strong distance-dependent mechanism for the quantification of biomolecular distances and concentrations *via* fluorescent biosensing. Genetically encoded FRET partners undergo conformational changes upon phosphorylation.^26,27^ The distance of the donor and the acceptor is related to the efficiency of FRET. While FRET assays are convenient tools for continuous monitoring of peptidases, their application to kinase assays typically relies on indirect readouts.^4,52–56^ Several fluorescent analogues for FRET have been developed and in theory can be applied for kinase sensing. However, most FRET changes are less than 50% which greatly reduces the sensitivity of these probes and kinase activity has to be high to be detected.^14,57,58^

### Early chelation-enhanced fluorescence (CHEF) probes for kinase sensing

**Fig. 2:**
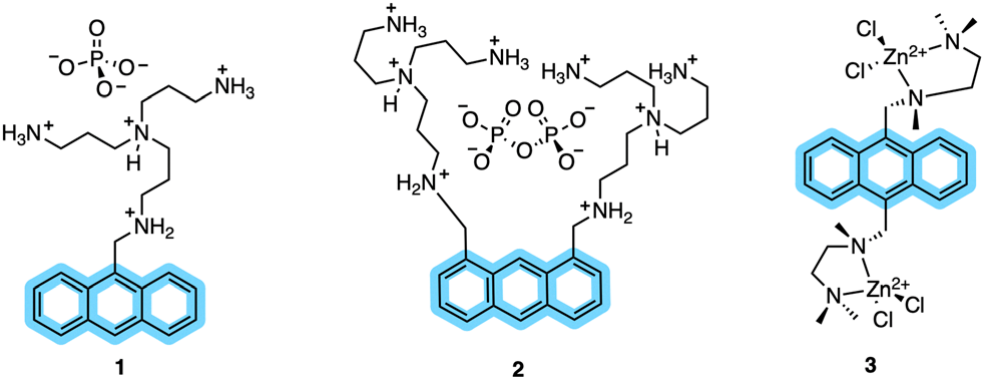
Early CHEF probes (1–3) developed by Czarnik and co-workers. Anthracene moieties conjugated with polyamine binding sites yielded strong CHEF “ON” responses in organic and aqueous media.

In kinase-sensor design, CHEF probes have emerged as a class of modular scaffolds. These probes have no fluorescence (or very weak fluorescence) themselves but upon chelation with a metal ion, a significant fluorescence enhancement is observed.^24,59^ CHEF probes are distinguished by their heteroatoms (for example amines) containing a suitably positioned lone pair that quenches fluorescence through photoinduced electron transfer (PET) and thereby increases fluorescence.^10,60–63^ The coordination effectively stabilizes the energy of the lone-pair-derived orbital relative to the fluorophore, restoring emission.^64^ Early CHEF probes for Zn^2+^ sensing and in turn inorganic phosphate detection were developed by Huston and co-workers. They synthesized a structurally simple anthracene fluorophore (1) that reported dramatic fluorescence increase (over 1000-fold) upon Zn^2+^chelation in acetonitrile.^65^ Over the next several years the same group developed additional anthrylazamacrocycle conjugate probes to explore their CHEF properties.^66^ Probes 2 and 3 (Fig. 2) showed remarkable fluorescence enhancements (from 108- up to 140-fold) by addition of Zn^2+^ and Cd^2+^ in aqueous media.^67^ In 1994, Vance and Czarnik established the first non-destructive, real-time assay using a high affinity anthrylpolyamine CHEF chemosensor (3). This probe showed micromolar affinity for pyrophosphate, over 2000-fold higher than for phosphates, allowing direct monitoring of inorganic phosphatase.^68^ Although potent, probes 1–3 lacked specificity and selectivity. These sensors could not be translated to kinase monitoring, leaving an innovation gap until the late 1990s, when the Imperiali group pioneered the use of SOX-labelled peptides for monitoring kinase activity.^12,14,69,70^ This offered considerable scope for the development of self-sensing sensors for assaying kinase activity in real-time.^2,14,71,72^

### SOX-labelled peptides for continuous kinase assays

**Scheme 1:**
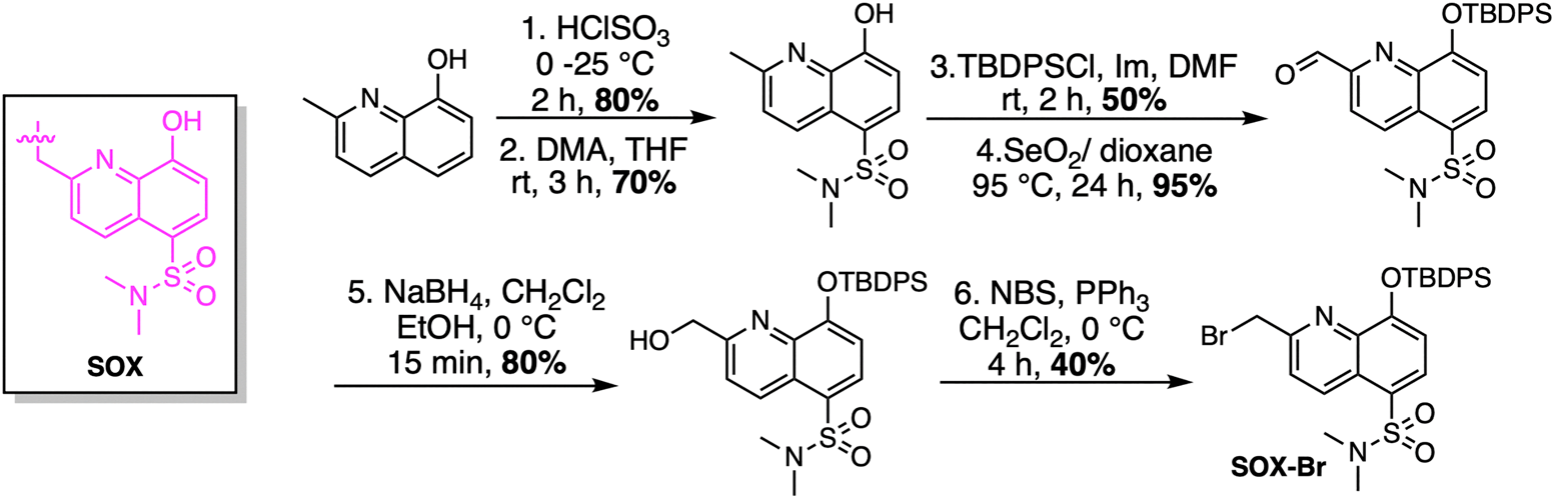
Synthesis of SOX-Br. DMA: dimethylamine, TBDPSCl: *tert*-butyldiphenylsilyl ether, Im: imidazole, NBS: *N*-bromosuccinimide.

Given the ability of anthracene probes to chelate phosphate anions and Zn^2+^, chelation-sensitive fluorophores were soon incorporated into peptides to create self-sensing kinase sensors. The modular design of these peptides relies on a kinase recognition motif, tagged with the phosphorylation-sensitive SOX fluorophore (Scheme 1).^14,15,73^ Unlike conventional fluorophores, SOX (*λ*_ex_ =365 nm and *λ*_em_ =485 nm) reports direct readouts of kinase activity in their native chemical environment.^14^ SOX is covalently attached to a peptide substrate and utilized to report tyrosine or serine/threonine phosphorylation *via* the CHEF effect.^70,74^ SOX-peptides have low fluorescence but upon phosphorylation and chelation to Mg^2+^, a large increase in fluorescence is observed due to the CHEF effect.^73^ The SOXylating agent required for the introduction of the SOX fluorophore into peptides, SOX-Br, is synthesized from commercially available 8-hydroxy-2-methylquinoline (Scheme 1). Chlorosulfonylation introduces a sulfonyl chloride group, followed by displacement of chloride with dimethylamine. This intermediate is then protected as a *tert*-butyldiphenylsilyl ether before the benzylic position is brominated *via* a Riley oxidation, reduction and bromination to yield the quinoline-based fluorophore SOX-Br.

**Fig. 3:**
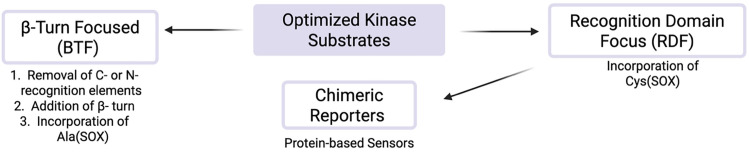
Distinct classes of SOX-peptide probes. Recognition elements are fully conserved in the RDF design and only partially so in the BTF design due to the required β-turn. The SOX chromophore can be positioned N- or C-terminal to the Ser/Thr/Tyr residue in both designs.

The Imperiali lab established two types of modular SOX sensors: (i) tunable Zn^2+^ sensors, which are beyond the scope of this review; and (ii) versatile probes of kinase activity. Three distinct classes of SOX-peptides have been profiled: (1) β-turn focused (BTF); (2) recognition-domain focused (RDF); and (3) chimeric.^2,29,69,73^ These peptide sensors have the SOX group incorporated within the nonnatural amino acids Ala(SOX) or Cys(SOX). The BTF-peptides have a β-turn motif containing Ala(SOX), and either an *N*- or *C*-terminal kinase recognition motif (Fig. 3).^14,15,75^ Although active under a variety of conditions, the BTF-peptides are limited by this conformationally constrained β-turn moiety.^12,69^ This separates the fluorophore from the phosphorylation site, rendering some sensors less selective and specific (*i.e.*, more promiscuous) due to loss of sequence recognition determinants.^15,76,77^ To address this challenge, a more powerful class of SOX-peptides was developed, the recognition-domain focused (RDF) peptides. In the RDF design, *S*-alkylation of a cysteine residue with SOX-Br affords Cys(SOX). This approach circumvents the constrained nature of β-turn motifs.^76^ With the aim of developing optimal sensors, the RDF strategy has been extended to protein-based probes, termed chimeric reporters. In chimeric reporters, the Cys(SOX) can be placed anywhere in the protein to discriminate challenging targets with short or ubiquitous consensus sequences.^78,79^

**Scheme 2:**
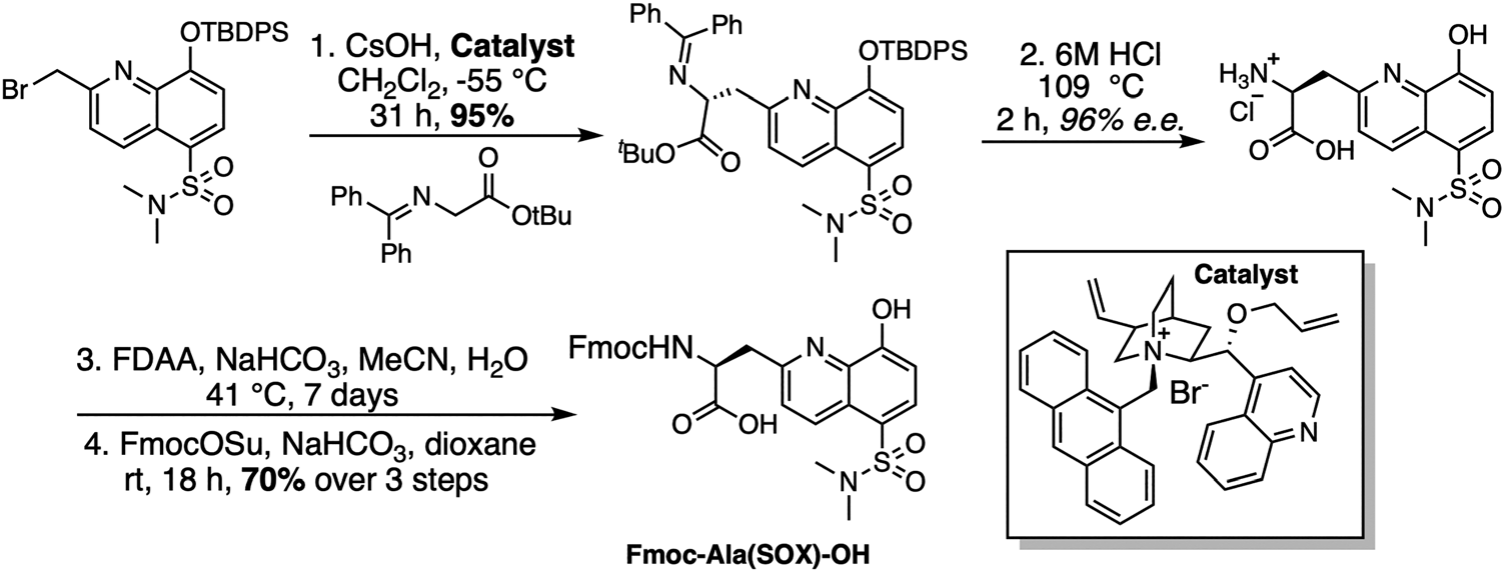
Synthesis of Fmoc-Ala(SOX)-OH for use in Fmoc SPPS. FDAA = 1-Fluoro-2-4-dinitrophenyl-5-l-alanine amide; Fmoc = 9-fluorenylmethyloxycarbonyl.

SOX-peptides and -proteins can be prepared using published methods.^80^ For the synthesis of the BTF class of SOX-peptides, the unnatural amino acid Fmoc-Ala(SOX)-OH was synthesized and used (Scheme 2).^14,70,74^ SOX-Br is used to alkylate a glycine derivative in the presence of a phase-transfer catalyst, which upon acid hydrolysis yields Ala(SOX). Fmoc protection is then carried out, yielding Fmoc-Ala(SOX)-OH for peptide synthesis.^69,70,74^

**Scheme 3:**
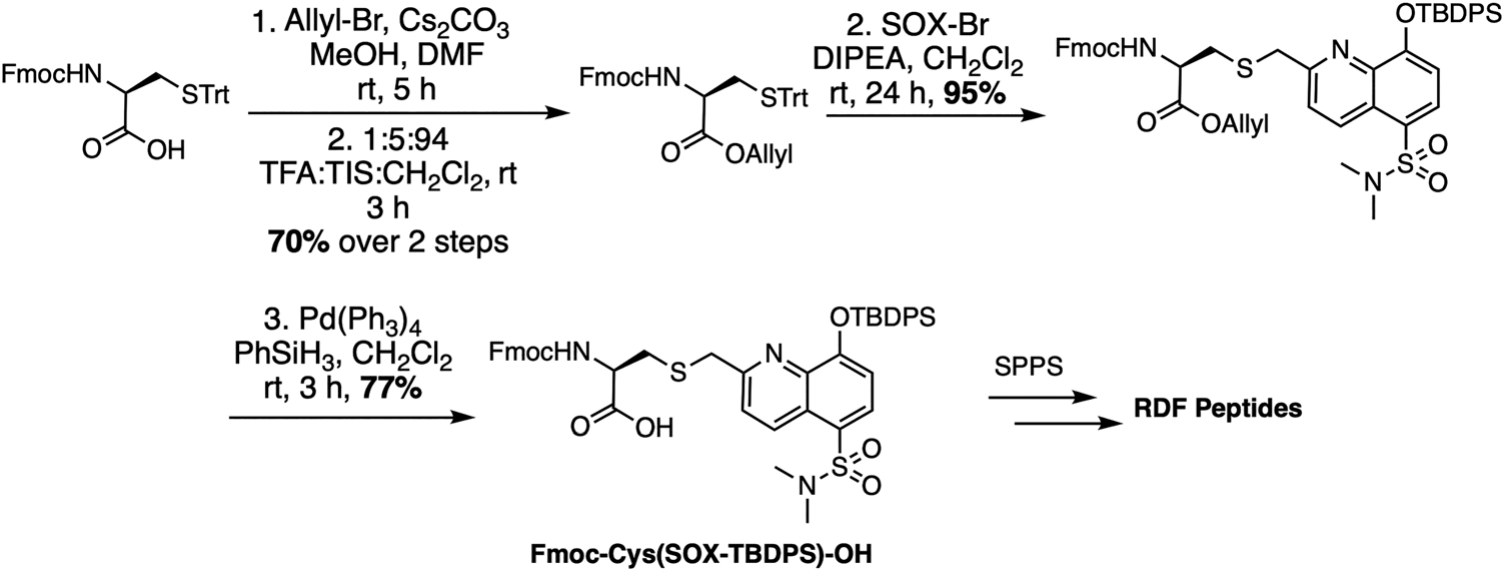
Synthesis of Fmoc-Cys(SOX(TBDPS))-OH for use in Fmoc SPPS. Trt: trityl, DIPEA: *N*,*N*-diisopropylethylamine, TFA: trifluoroacetic acid.

The RDF class of SOX-peptides are also constructed *via* Fmoc-based SPPS but rather than utilizing Fmoc-Ala(SOX)-OH, they were prepared using Fmoc-Cys(SOX(TBDPS))-OH (Scheme 3).^69^ This includes an appropriately placed cysteine protected with a trityl group. After selective on-resin sulfhydryl group deprotection, the free thiol is alkylated with SOX-Br. Standard TFA cleavage and concomitant removal of all the side chain protecting groups afford the non-natural Cys(SOX) amino acid with excellent conversion (>95%). Cys(SOX) is then utilized in automated SPPS or SPOT synthesis to generate RDF peptide libraries.^12,69,80^

### β-Turn focused (BTF) sensors

**Fig. 4:**
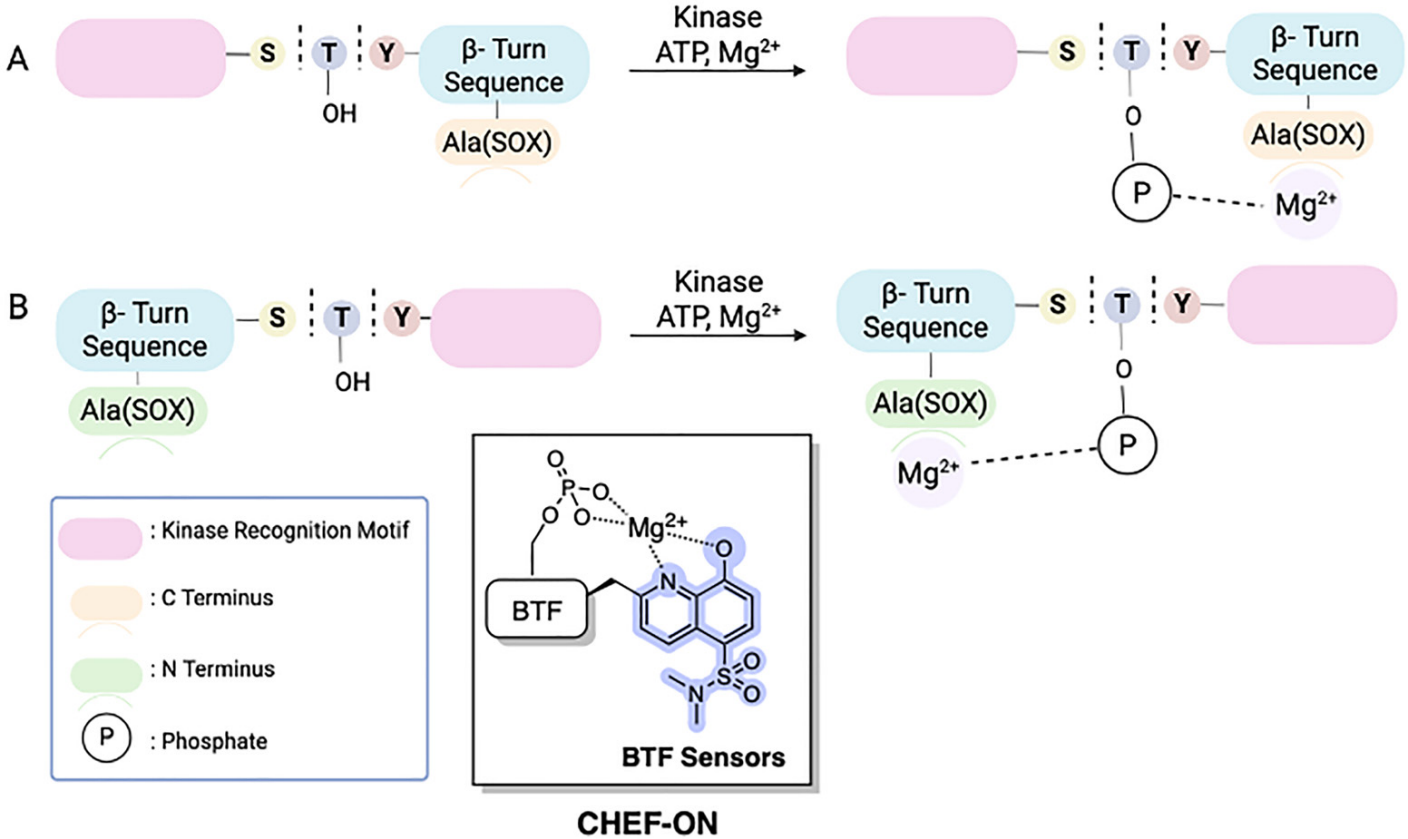
Schematic representation of 1st generation SOX-peptides, termed BTF sensors. The key modules include the SOX fluorophore, separated from the phosphorylation site by a β-turn. The binding site for Mg^2+^ is preorganized by the β-turn sequence. Design of kinase recognition motif for C-terminus (A) and N-terminus (B).^75^

BTF sensors have been successfully applied as chemosensors (with 4–12-fold fluorescence increases upon phosphorylation) to monitor kinase activity with pure enzyme and in unfractionated cell extracts.^76^ The BTF design contains Ala(SOX) (−)2 or (+)2 amino acids away from the phosphorylation site, a kinase recognition motif that includes the amino acid being phosphorylated, and a β-turn that preorganizes a binding site for interaction with Mg^2+^.^14,69,75,77^ The β-turn also prevents interference of the SOX moiety with the enzyme by placing it further away from the phosphorylation site (Fig. 4). The kinase recognition motif is varied to target the desired kinase. BTF sensors successfully targeted kinases including PKC, PKA, Akt and MK2 (Table 1).^14^ Notably, these kinase assays required less than 0.1 mg total cell protein for measurements compared to the ∼0.5 mg for complex kinase assays.^75^ Across the range of probes, serine yields larger increases in fluorescence than threonine (*cf.* PKC-S1 with PKC-S2 and Akt-S1 with Akt-S4). Another meaningful trend is that Mg^2+^ coordination is enhanced with rigidity (*cf.* Cdk2-S1 with PKC-S2 and PKA-S3 with Pim2-S2). Incorporation of an additional aspartate residue (*cf.* PKC-S5 and PKC-S6) didn’t significantly change the fluorescence compared to the closely related peptide PKC-S4.^69,76^

**Table 1:** Comparison of BTF and RDF SOX-peptides as kinase sensors

Target kinase	Design	Substrate sequence (all *C*-terminal amides)	*K*_M_ (µM)	Fold fluorescence increase
PKC_α_	BTF	Ac-Ala(SOX)-P-G-S̲-F-R-R-R	8.6 ± 2.9	5.7
PKA		Ac-L-R-R-A-S̲-L-P-Ala(SOX)	1.8 ± 0.5	1.3
Akt		Ac-A-R-K-R-E-R-A-Y-S̲-F-_D_P-Ala(SOX)-G	3.8 ± 0.1	9.3
MK2		Ac-A-H-L-Q-R-Q-L-S̲-I-_D_P-Ala(SOX)-G	21 ± 1	8.5
PKA		Ac-L-R-R-A-S̲-L-_D_P-Ala(SOX)-G	2.9 ± 0.3	5.6
PKC-S1		Ac-Ala(SOX)-P-G-S̲-F-R-R-R	8.6 ± 2.9	6.7 ± 0.6
PKC-S2		Ac-Ala(SOX)-P-G-T̲-F-R-R-R	23 ± 2.3	3.8
Cdk2-S1		Ac-Ala(SOX)-P-K-T̲-P-K-K-A-K-K-L	0.6 ± 0.1	4.7 ± 0.5
PKA-S2		Ac-L-R-R-A-S̲-L-P- Ala(SOX)	1.8 ± 0.5	4.0
PKA-S3		Ac-L-R-R-A-S̲-L-_D_P- Ala(SOX)-G	2.9 ± 0.6	6.5 ± 0.7
Akt-S1		Ac-A-R-K-R-E-R-A-Y-S̲-F-_D_P-Ala(SOX)-G	3.8 ± 0.2	7.6 ± 0.6
Akt-S4		Ac-A-R-K-R-E-R-T-Y-T̲-F-_D_P-Ala(SOX)-G	3.8 ± 0.7	4.5± 0.3
MK2-S1		Ac-A-H-L-Q-R-Q-L-S̲-I- _D_P-Ala(SOX)-G	21 ± 2	7.7 ± 0.9
Pim2-S2		Ac-A-R-K-R-R-R-H-_D_P-S̲-G-P-Ala(SOX)-G	8.6 ± 2.9	3.0 ± 0.4
Src	RDF	Ac-A-E-E-Cys(SOX)-I-Y̲-G-E-F-E-A-K-K-K-K	7.0 ± 1.0	2.0 ± 0.1
Abl		Ac-E-Cys(SOX)-I-Y̲-A-A-P-F-A-K-K-K	10.5 ± 1.5	4.6 ± 0.5
IRK		Ac-R-Cys(SOX)-D-Y̲-Nle-T-M-Q-I-G-K-K	27.1 ± 3.9	4.2 ± 0.1
PKC_α_		Ac-R-R-R-Cys(SOX)-A-S̲-F-R-R-R	0.13 ± 0.02	3.7 ± 0.2
PKC_βI_		Ac-L-K-R-Cys(SOX)-A-S̲-F-K-K-F-A	0.81 ± 0.18	9.7 ± 0.5
PKC_δ_		Ac-R-K-R-K-G-S̲-F-Cys(SOX)-Y-G-G	0.48 ± 0.07	7.3 ± 0.1
Pim2		Ac-A-R-K-R-R-R-H-P-S̲-G-Cys(SOX)-P-T-A	1.4 ± 0.1	3.2 ± 0.1
Akt1		Ac-A-R-K-R-E-R-A-Y-S̲-F-Cys(SOX)-H-H-A	0.69 ± 0.11	3.9 ± 0.3
MK2		Ac-A-H-L-Q-R-Q-L-S̲-I-Cys(SOX)-H-H	1.2 ± 0.2	4.4 ± 0.2
PKA		Ac-A-L-R-R-A-S̲-L-Cys(SOX)-A-A	2.6 ± 0.3	5.0 ± 0.2

### Recognition-domain focused (RDF) sensors

**Fig. 5:**
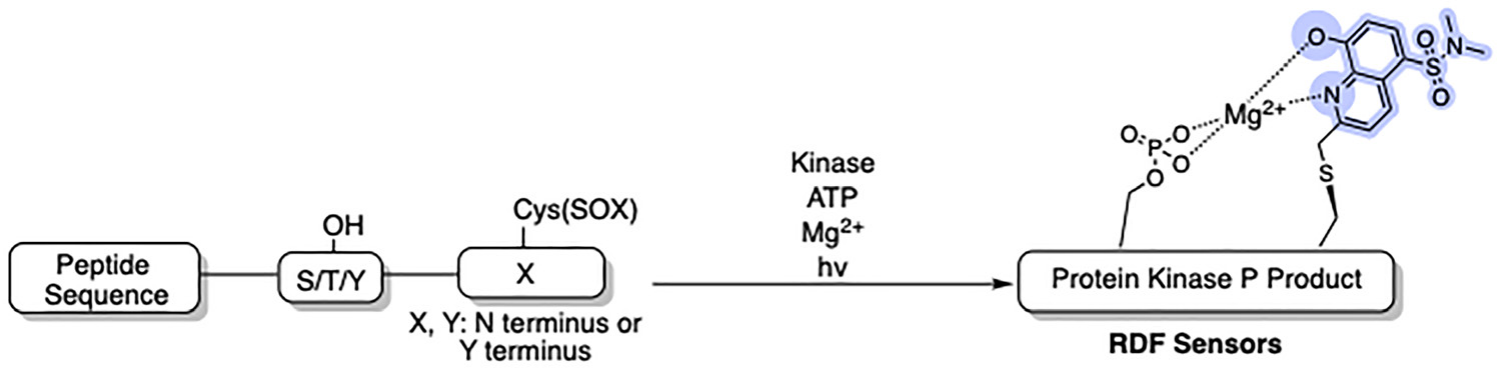
RDF designs from optimized nonfluorescent substrates. Circumventing the β-turn requirement allows the recognition elements to be fully conserved in the RDF design. The SOX chromophore can be positioned N- or C- terminus to the Ser/Thr/Tyr residue.^77^

RDF-sensors enabled the inclusion of extended binding sequences, which maximized recognition by the cognate kinase (Fig. 5). Cys(SOX) is placed either (−)2 or (+)2 amino acids from the phosphorylation site, similarly to BTF-peptides. RDF-peptides offer several advantages compared to BTF peptides: (i) kinase recognition sequences can be included on both sides of the phosphorylation site; (ii) SOX addition to cysteine can be performed either during SPPS or on unprotected peptides *via* cysteine SOXylation; (iii) up to 28-fold improved catalytic activity; and (iv) improved kinetic parameters (up to 66-fold lower *K*_M_ compared to their BTF counterparts).^69,77^ A variety of different RDF-peptides have been constructed that target representative Ser/Thr (PKC_α_, PKC_βI_, PKC_δ_, Pim2, Akt1, MK2 and PKA), receptor (IRK) and nonreceptor Tyr (Src, Abl) kinases.^77^ Based on the RDF design, new SOX-peptides have been developed using high-throughput mass-spectrometry.^12^ Furthermore, these RDF-peptides were used in a 384 well plate format to monitor the activity of multiple kinases at once (multiplexing). This shows the amenability of SOX probes for the screening of kinase inhibitors, with the potential for such inhibitors to be taken forward as therapeutic leads. Indeed, the Dalby laboratory detailed the use of SOX-based sensors to identify eEF-2 inhibitor scaffolds from a 32 960-member inhibitor library.^81,82^ The “SOXtide” substrate (Ac-RKK**Y̲**KFNC-Cys(SOX)-DRRRFL-NH_2_) resulted in a five-fold increase in fluorescence emission. This allowed for the continuous monitoring of eEF-2 activity *in vitro*, including samples of tissue homogenates and lysates.^81^ Stains and colleagues described three optimal sensor constructs (FAKtide-S2, Nictide-S2 and ROCK-S1) for focal adhesion kinase (FAK), leucine-rich repeat kinase 2 (LRRK2) and Rho-associated protein kinase (ROCK) respectively.^83–86^ FAKtide-S2 (Ac-VSETDD**Y̲**A-Cys(SOX)-IIDEEDT-NH_2_) and Nictide-S2 (Ac-RLGWWR-Cys(SOX)-YT̲LRRARQGNTKQR-NH_2_) detected concentrations of their targets as low as 1.0 and 2.5 nM and are highly reproducible (Z’ = 0.91 and 0.7 respectively). Similarly, ROCK-S1 (KPARKKRYT̲V-Cys(SOX)-GNPYWM) sensitively reported ROCK activity with a limit of detection of 10 pM. As a proof-of-principle ROCK-S1 also rapidly identified inhibitor scaffolds (PHA665752 and IKK16) against ROCK2 from a 78 small molecule library. FAK, and ROCK are actively associated with hepatocellular carcinoma tumors (HCC), while LRRK2 with forms of Parkinson's disease.^87–89^

### Chimeric sensors

**Fig. 6:**
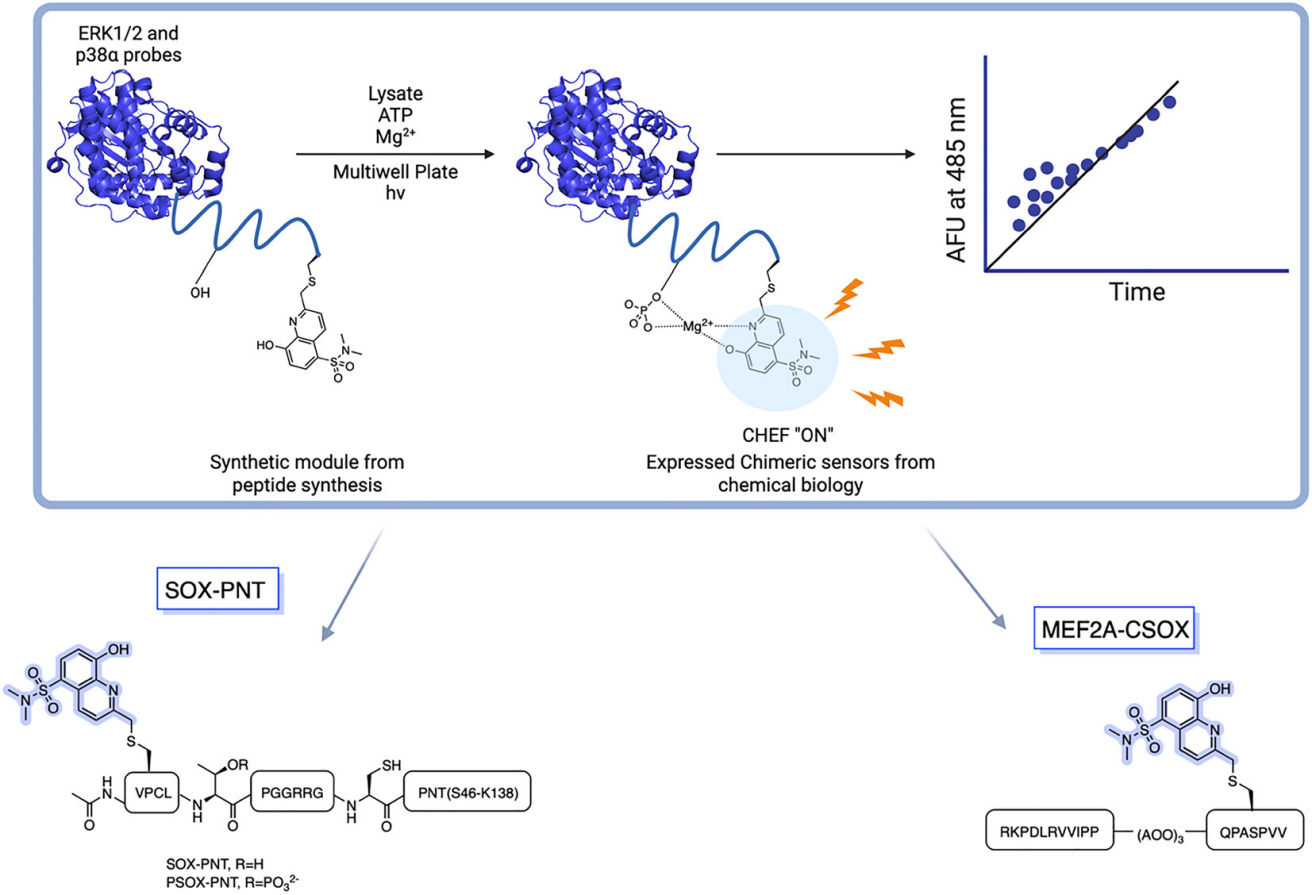
Rational design of Cys(SOX)-based chimeric sensors SOX-PNT and MEF2A-CSOX in which a known peptide sequence is linked to CSOX *via* a flexible linker. SOX-PNT probes the ERK1/2, utilizing the PNT domain from Ets-1 for specific binding to the enzyme. The docking domain motif of MEF2A-CSOX is linked to the phosphorylation site with AOO linker.

BTF- and RDF-sensors gave sensitive and robust signals with direct readouts under physiological conditions when the SOX group is placed within (+2) or (−2) amino acids away from the phosphorylation site.^76,77^ However, several physiologically important kinases including mitogen-activated protein kinases (MAPKs) are acutely reliant on secondary interactions distal to the phosphorylation site that impart substrate specificity.^2,15,76,78,79^ MAPKs are extremely challenging in terms of sensor design due to the minimal consensus sequences required for recognition, such as a Ser-Pro or a Thr-Pro dipeptide motif.^2,78^ To achieve higher selectivity for MAPKs, the third generation of SOX biosensors was developed, known as chimeric sensors. These are protein-based reporters, where the Cys(SOX) is placed anywhere in the protein, enabling the detection of challenging targets with short or ubiquitous consensus sequences.^90^ Two chimeric sensors were reported for ERK1/2 and p38-α, named SOX-PNT and MEF2A-CSOX respectively (Fig. 6). SOX-PNT consists of the recombinant PNT domain from the Ets-1 transcription factor and the synthetic ERK1/2 consensus motif.^79,91–93^ In MEF2A-CSOX the phosphorylation site is linked to the docking peptide sequence *via* a flexible linker AOO (8-amino-3,6-dioxaoctanoic acid). Both probes showed good affinities (*K*_MSOX-PNT_ = 14.9 µM and *K*_MMEF2A-CSOX_ = 1.3 µM) alongside excellent fluorescence responses (4–17-fold) which would be impossible to achieve with simple peptide probes.^78,79^ Background affinity is minimal in the presence of closely related isoforms.^78,79^ Using a docking domain-based strategy, three activity sensors were developed for the kinase subfamilies, ERK1/2, p38α/β and JNK1/2/3 (Table 2). Each manifested a 70-fold enhanced affinity. Triazole and polyethylene glycol were used as linkers to join the two sensor modules. The three chimeric sensors showed improved binding affinities (*K*_M_ (*ca* 0.14–3.3 µM)) compared to SOX-PNT and MEF2A-CSOX. These MAP kinase sensors quantified kinase activities in cell lysates over an extended time course. Kinase inhibitor activities were also monitored in either recombinant protein or cell-lysate based assays.^2^ The MAP sensors were also applied *in vitro* in a breast cancer progression model based on the MCF10A series, where p38α/β activity correlated with increased tumorigenicity.^2,13,94–96^ This further underscored the practical utility of SOX-chimeric sensors in profiling kinase activity and quantifying disease progression in cell culture models.

**Table 2:** Sequences of Modular MAPK Activity Sensors

Kinase	Docking motif	Linker	Phosphorylation site
JNK1/2/3	ERPSRDHLYLPLEP	PEG_2_	SANLLS̲P̲-Cys(SOX)-PA
p38α/β	IKIKKIEDASNPLLLKRRKKG	PEG_2_-Triazole- PEG_2_	QP-Cys(SOX)-AS̲P̲VV
ERK1/2	PQLKPIESSILAQRRVRKLG	PEG_2_-Triazole- PEG_2_	VP-Cys(SOX)-LT̲P̲GGRR
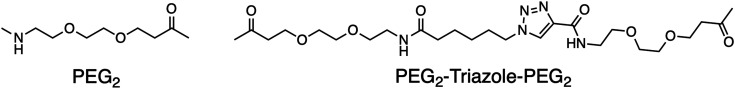			

### SOX peptides for differential sensing of MAPKs

**Fig. 7:**
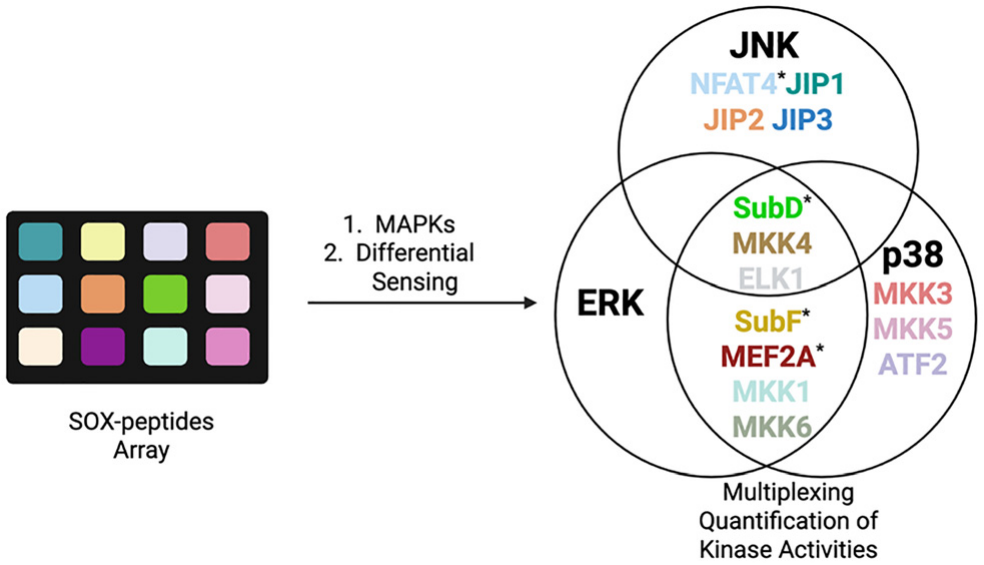
Multiplexing of MAP kinase activity with 14 SOX-peptides, using differential sensing. The peptides of the original array are denoted with an asterisk.

While RDF and chimeric sensors reported accurate kinase readouts *via* pre-organized Mg^2+^ chelation, discrimination among closely related kinases, including MAPKs, remains challenging. Conversely, an array of SOX peptides can fingerprint kinase isoforms *via* a single pattern recognition approach, known as differential sensing (DS).^27,97,98^ Anslyn *et al.* reported five SOX peptides with distinctive docking sites and selectivities for MAPK families that contain several closely related isoforms. These display *ca* 80% identity over the catalytic domains. Their array differentiated each of the following families at nanomolar levels (0.5–5 nM) from a single pattern response: ERK1/2, JNK1/2/3 and p38α/β/δ. The discriminatory power of differential sensing was further validated by qualitatively detecting specific inhibitor concentration in one *in vitro* assay. In 2022, the same team followed the activity of numerous kinases at once (multiplexing) using DS. Retaining the use of four SOX-peptides, they expanded the original array to a much broader one of 14 SOX-peptides in total (Fig. 7). Using machine learning (*e.g.* principal component analysis (PCA)) they achieved excellent discrimination of nine isoforms of JNK and p38 families. In the DS array, ERK isoform displayed the poorest discrimination, consistent with their 87–88% similar identity. Notably, this viable quantitation method successfully multiplexed the three kinase activities in A549 cell lysates, albeit with increased errors.^27,97^

### Rationally designed SOX-peptides for interrogating Arg kinase and phosphatase

**Fig. 8:**
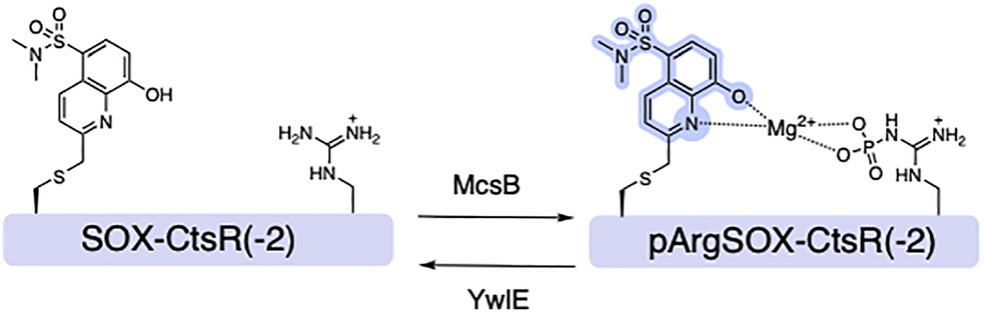
Design of SOX-CtsR(−2) sensor for bacterial Arg Kinase McsB and the cognate phosphatase YwlE, and representative spectra of SOX-CtsR(−2) and the phosphorylated form pArgSOX-CtsR(−2) in the presence of Mg^2+^.

While most studies have been performed on protein alcohol (Ser/Thr/Tyr) kinases, protein guanidine (*e.g.*, Arg) kinases also exist.^99–102^ In Gram-positive bacteria arginine phosphorylation is regulated by Arg kinase McsB and the cognate phosphatase YwlE, which are crucial regulators of stress responses and pathogenesis.^103,104^ In response to heat stress, McsB phosphorylates arginine to produce phosphoarginine (pArg).^105,106^ To probe both McsB and YwlE, Kee and coworkers reported SOX-CtsR(-2), a reversible SOX-peptide that reports a five-fold fluorescence increase upon phosphorylation.^107^ Notably, in the presence of YwlE both the Arg phosphorylation level and fluorescence intensity decreased, validating the dual function of SOX-CtsR(-2) (Ac-KVIQ-Cys(SOX)-K**R̲**GGGGYIK-NH_2_) (Fig. 8). Arg phosphorylation is dynamically controlled by the opposing actions of McsB and YwlE. Yet, SOX-CtsR(-2) suggests that McsB mediates the dephosphorylation of the pArg residue to generate ATP.^107,108^ SOX-CtsR(-2) is the first example of a SOX-peptide used to study the interplay between McsB and YwlE. Using this sensor, it was demonstrated that McsB kinetic activity is modulated by factors including autophosphorylation state and nucleotide levels. While counterintuitive, this study revealed that McsB can dephosphorylate pArg even without the presence of YwlE. In addition to these applications, SOX-peptides can also provide insights into such complex mechanisms that until now remained elusive.

**Fig. 9:**
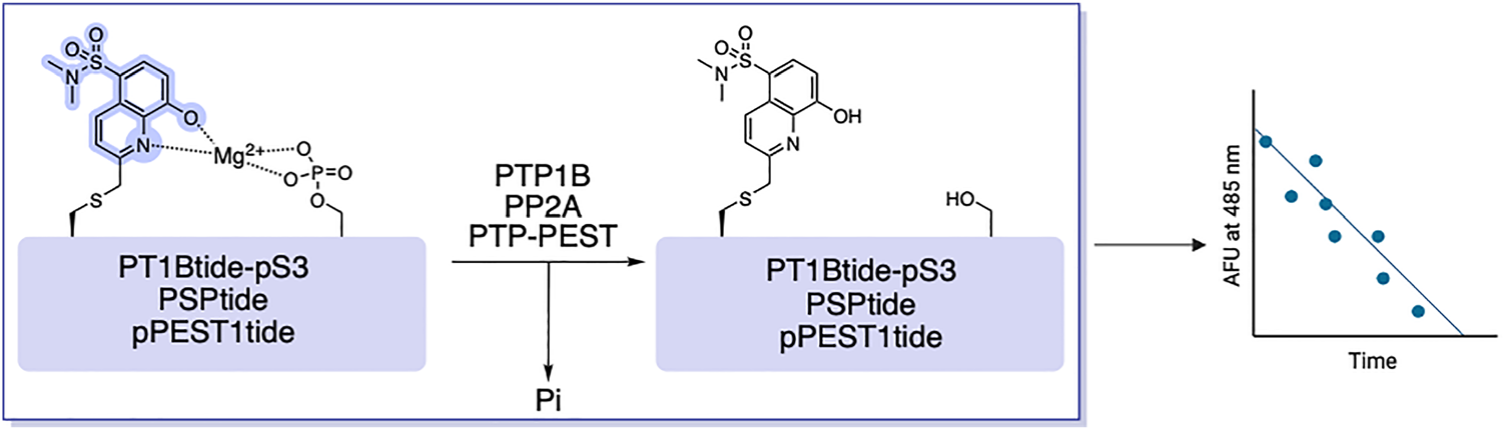
Reported SOX-based protein phosphatase sensors. Phosphatase activity removes the phosphate group from the peptide probe and greatly reduces the affinity for Mg2+ leading to a decrease in fluorescence.

Compared to the >500 known kinases, the human genome encodes only ∼30 protein serine/threonine phosphatases (PPs), which have been referred to as the “ugly ducklings” of cellular signalling.^6,106,109^ However, increasing amounts of evidence suggests that dysregulation of phosphatases can play a critical role in human disease states, including hepatocellular carcinoma (HCC) and the metabolic disorder known as NAFLD.^110–113^ Being able to continuously monitor their enzymatic activity *via* fluorescent readouts is desirable for mechanistic investigations. For this purpose, the Stains lab demonstrated that SOX-peptides can be repurposed to afford direct activity assays for protein phosphatases.^114,115^ Dephosphorylation of the CHEF sensor by a phosphatase results in a decrease in the fluorescence over time, which is directly proportional to the activity of the target enzyme (Fig. 9). The first proof-of principle probe termed PT1Btide-pS3 (ARDI-**p̲Y̲**-R-Cys(SOX)-FFRKG), was based on the autophosphorylation sequence of ALK, a known substrate for protein tyrosine phosphatase-1B (PTP1B).^114,116,117^ PT1Btide-pS3 can detect as little as 12 pg full-length recombinant PTP1B in a remarkably selective trend.^114^ This optimal chemosensor also provided clear evidence for elevated PTP1B catalytic activity in a rat model of NAFLD disease that afflicts 20–30% of the US population.^115^ The generality of this approach was further investigated with the PSPtide sensor (Ac-DRRV-**pS**-V-Cys(SOX)-NH_2_).^118,119^ Utilizing this sensor the temporal dynamics of protein phosphatase-2A (PP2A) activity during insulin stimulation of liver hepatocytes were profiled.^120–122^ Background subtraction of any off-target serine/threonine phosphatase activity using calyculin A allowed isolation of inhibitor-sensitive phosphatase activity.^114,116,123,124^ Assignment to PP2A was further supported by borthogonal validation through siRNA knockdown and immunodepletion, which reliably quantified PP2A activity in unfractionated cell lysates.^116^ This validated assay demonstrated a significant modulation of PP2A activity across a panel of human carcinoma cell lines (HCT116, HeLa and HepG2).^114^ Another Cys(SOX)-based phosphatase CHEF probe is the pPEST1tide (Ac-YDEDF**pY**D-Cys(SOX)-EF-NH_2_) for monitoring the phosphatase non-receptor type 12 (PTPN12 or PTP-PEST) activity.^125^ Gratifyingly, decreasing concentrations of PTP-PEST yielded a limit of detection of 0.2 nM with the pPEST1tide. This level of sensitivity is comparable with PT1Btide-pS3 and PSPtide. However, the applicability of pPEST1tide in interrogating the role of PTP-PEST in HCC and other aggressive tumors remains underexplored.^111,125,126^

### SOX-peptide technologies now commercially available

SOX-peptides are now commercially available through the Phosphosens® platform from AssayQuant Technologies Inc. (https://www.assayquant.com), who have developed a set of validated assay formats for a broad range of kinases and phosphatases. Their standardized continuous kinetic assays allow direct monitoring of enzyme activity in real time, without the need for coupling enzymes, antibodies, or wash steps, and can be performed on purified recombinant enzymes and cell and tissue lysates. In addition, they have developed a red-shifted “PhosphoSens-Red” format that uses Eu^3+^ salts to generate a red-shifted time-resolved fluorescence, which helps reduce background interference and compound autofluorescence. However, because Eu^3+^ is introduced after the kinase reaction, this format converts the assay to an endpoint readout rather than a continuous one, improving practicality for screening while sacrificing the real-time kinetic information available from the original Mg^2+^-dependent CHEF format.

## Conclusions and future outlooks

This review has described how SOX-peptides allow direct, continuous measurement of kinase and phosphatase activity, providing access to kinetic information that is difficult to obtain using conventional endpoint assays. In appropriate systems, these tools offer a practical alternative to antibody- and radioactivity-based methods, particularly for studies in unfractionated lysates and inhibitor screening. Despite these advantages, CHEF probes have several important limitations. The synthesis of SOX-labelled substrates and related fluorophores is not trivial as it involves multiple steps, fragile phenol protecting groups, limited overall yields and often challenging workup and purification steps due to product solubility and sensitivity. Thus, custom SOX probes are not easily accessible and large-scale preparation remains demanding for laboratories without expertise in chemical synthesis. In addition, current CHEF sensors require chemical synthesis by SPPS, which is resource-intensive and generates substantial solvent and reagent waste. Unlike genetically encoded FRET reporters, SOX-peptides have yet to be generated inside cells, limiting their use in long-term or spatially resolved live-cell studies. From an optical perspective, SOX-based probes are also constrained by their short excitation wavelengths (typically 360–365 nm), which increases background interference and phototoxicity in biological samples. For phosphatase assays, additional complications can arise from enzyme sensitivity to oxidation, which can introduce artefacts unless strictly controlled. Several development directions address these issues. One approach is the incorporation of red-shifted fluorophores or lanthanide complexes to enable excitation in the visible range and improve signal-to-noise ratios. Time-resolved fluorescence (TRF) formats using lanthanides (*e.g.* Tb^3+^, Eu^3+^) further reduce background interference by red-shifting the detection wavelength. Another avenue is the design of alternative Mg^2+^-responsive chromophores that retain the CHEF mechanism but exhibit more favorable photophysical properties. Progress in this area has been limited over the past decade, with relatively few new scaffolds beyond SOX and closely related analogues. Overall, CHEF probes represent a robust platform for real-time enzymatic assays, particularly in biochemical and screening contexts. Broader adoption of lysate-compatible assays can accelerate discovery, profiling and optimization.

## Conflicts of interest

There are no conflicts to declare.

## Abbreviations

Abl: Abelson kinase
ADP: Adenosine diphosphate
Akt: Protein kinase B
ALK: Anaplastic lymphoma kinase
AOO: 8-amino-3,6-dioxaoctanoic acid
ATP: Adenosine triphosphate
BTF: β-turn focused
Cdk: Cyclin-dependent kinase
CHEF: Chelation-enhanced fluorescence
DS: Differential sensing
eEF-2: Eukaryotic elongation factor 2
EGFR: Epidermal growth factor receptor
ERK: Extracellular signal-regulated kinase
FAK: Focal adhesion kinase
FDAA: 1-fluoro-2,4-dinitrophenyl-5-l-alanine amide
Fmoc: 9-fluorenylmethoxycarbonyl
FRET: Förster resonance energy transfer
HCC: Hepatocellular carcinoma
HTS: High-throughput screening
IRK: Insulin receptor kinase
JNK: c-Jun N-terminal kinase
*K*_M_
: Michaelis constant
LOD: Limit of detection
LRRK2: Leucine-rich repeat kinase 2
MAPK: Mitogen-activated protein kinase
McsB: Protein arginine kinase McsB
MEF2A: Myocyte enhancer factor 2A
MK2: MAPK-activated protein kinase 2
NAFLD: Non-alcoholic fatty liver disease
NBS: *N*-bromosuccinimide
PCA: Principal component analysis
PEG: Polyethylene glycol
PET: Photoinduced electron transfer
pArg: Phosphoarginine
PHA665752: A small-molecule kinase inhibitor
Pim2: Proviral integration site for Moloney murine leukemia virus 2 kinase
PK/PD: Pharmacokinetic/pharmacodynamic
PKA: Protein kinase A
PKC: Protein kinase C
PP: Protein phosphatase
PP2A: Protein phosphatase 2A
PTM: Post-translational modification
PTP1B: Protein tyrosine phosphatase 1B
PTPN12: Protein tyrosine phosphatase non-receptor type 12
RDF: Recognition-domain focused
ROCK: Rho-associated protein kinase
SH2: Src homology 2
SOX: Sulfonamido-oxine
SPPS: Solid-phase peptide synthesis
Src: Proto-oncogene tyrosine-protein kinase Src
TBDPS: *Tert*-butyldiphenylsilyl
TDI: Time-dependent inhibition
TFA: Trifluoroacetic acid
Trt: Trityl
YwlE: Arginine phosphatase YwlE

## Data Availability

No new data were generated or analyzed in this study.
